# Optomechanic Coupling in Ag Polymer Nanocomposite Films

**DOI:** 10.1021/acs.jpcc.1c04549

**Published:** 2021-06-30

**Authors:** Adnane Noual, Eunsoo Kang, Tanmoy Maji, Manos Gkikas, Bahram Djafari-Rouhani, George Fytas

**Affiliations:** † Faculté Pluridisciplinaire Nador, LPMR, Université Mohammed Premier, Oujda BP 717-60 000, Morocco; ‡ Max Planck Institute for Polymer Research, Ackermannweg 10, Mainz 55128, Germany; § Department of Chemistry, University of Massachusetts Lowell, Lowell, Massachusetts 01854, United States; ∥ Institut d’Électronique, de Microélectronique et de Nanotechnologie (IEMN), UMR-CNRS 8520, Department of Physics, University of Lille, Villeneuve d’Ascq 59655, France

## Abstract

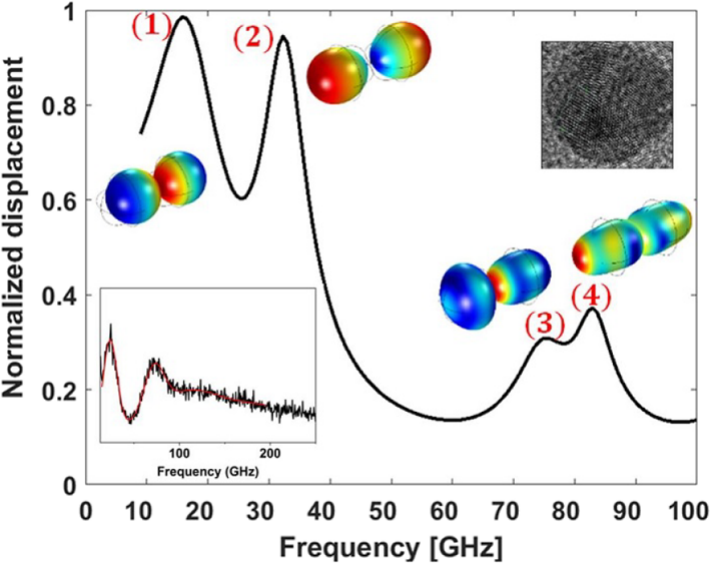

Particle vibrational spectroscopy has emerged as a new tool for the measurement of elasticity, glass transition, and interactions at a nanoscale. For colloid-based materials, however, the weakly localized particle resonances in a fluid or solid medium renders their detection difficult. The strong amplification of the inelastic light scattering near surface plasmon resonance of metallic nanoparticles (NPs) allowed not only the detection of single NP eigenvibrations but also the interparticle interaction effects on the acoustic vibrations of NPs mediated by strong optomechanical coupling. The “rattling” and quadrupolar modes of Ag/polymer and polymer-grafted Ag NPs with different diameters in their assemblies are probed by Brillouin light spectroscopy (BLS). We present thorough theoretical 3D calculations for anisotropic Ag elasticity to quantify the frequency and intensity of the “rattling” mode and hence its BLS activity for different interparticle separations and matrix rigidity. Theoretically, a liquidlike environment, e.g., poly(isobutylene) (PIB) does not support rattling vibration of Ag dimers but unexpectedly hardening of the extremely confined graft melt renders both activation of the former and a frequency blue shift of the fundamental quadrupolar mode in the grafted nanoparticle Ag@PIB film.

## Introduction

In the last decade, particle vibrational spectroscopy has emerged as a new tool for the measurement of elasticity, glass transition, and interactions at a nanoscale. For spherical dielectric particles in the submicron range in air, numerous hypersonic vibrations characterized by radial (*n*) and angular (*l*) momentum have been resolved by frequency domain Brillouin light spectroscopy (BLS).^1,2^ For ellipsoids in air, the lifting of degeneracy (*m* = 2*l* + 1) led to a complex experimental spectrum with increasing aspect ratios of random oriented samples.^3^ The vibration spectrum is also very sensitive to the presence of interparticle interactions which are manifested by the appearance of a new low frequency mode, termed the interaction mode, and a split and concurrently blue shift of the (1,2) mode.^4^ This spectral modification has been already utilized in granular nanomaterials.^5−8^ However, the application horizon is restricted in colloid-based materials in air as their infiltration by either liquid or solid medium renders the strong resonances (in air) weakly localized and hence hardly resolved.

The required enhancement was realized in metallic nanoparticles (NPs) by means of strong optomechanical coupling. The first reports were on an assembly of nanometer size Ag and Au NPs on solid substrates (graphite, Al_2_O_3_) using Raman spectroscopy.^9−12^ Subsequently, combination of SEM and picosecond acoustic (pump-probe) technique (PA) enabled probing of the elastic vibration for a single Au (diameter 85 nm) and a dimer Au NP on a glass substrate.^13^ The latter displayed a new mode, absent in the single Au NP, at a lower frequency than the spheroidal (1,2) mode also present in the single NP. The new mode, termed^13^ stretching, bears resemblance to the interaction mode in dielectric NPs.^4,7^ Several years later, BLS was utilized to record the vibration spectrum of an assembly of Au NPs for different diameters (D between 12 and 100 nm) in a glassy poly (vinylpyrrolidone) (PVP) matrix. The spectrum revealed the quadrupolar (1,2) mode and, at lower frequencies, the stretching mode (or quasi-translation).^14^ The latter associated with Au dimers was resolved with a 647 nm laser light being closer to the longitudinal plasmon resonance mode of the Au dimer than the 532 nm laser underlying the resonance enhancement. The well-established scaling, *f* (1,2) ∼ *c*_t_*D*^–1^, of the quadrupolar mode in PVP conforms to the scaling in air with the transverse sound velocity, *c*_t_ = 1250 m/s, of elastically isotropic Au. However, the frequency of the quasi-translational mode depends on the matrix elasticity as supported by eigenmode 2D (axial) calculations.^15,16^

Few reports focused on the vibration modes of sizeable (100–200 nm) Au NPs in PVP or in supported nanodevices utilizing BLS.^14,17,18^ and PA,^13,19−21^ respectively. The utility of large (∼100 nm) Au NPs in PVP allowed selective recording of single Au and Au dimer NPs using two lasers at 532 and 647 nm (polarized along the dimer long axis), respectively. For an isolated Au dimer, the single quasi-translation mode in an ensemble of Au NPs is split into two out-of-phase vibrations of the Au spheres along and perpendicular to the dimer axis, while the quadrupolar mode (∼14 GHz) is blue-shifted about 2 GHz with respect to the single Au NP.^14^ Both effects on the dimer frequencies were attributed to the hybridization of *l* = 1 and *l* = 2 modes of two Au monomers in close proximity. A further focus on the high frequency modes of a single Au dimer probed by 647 nm laser light polarized along its long axis was achieved.^17^ Aside from the blue shift of the *l* = 2 quadrupolar mode of an individual Au NP, additional Lamb modes with *l* > 2 were resolved in the BLS spectrum with decreasing interparticle separation.^18^ Eigenmode frequency and intensity calculations were based on the local variation of the electronic susceptibility caused by density fluctuations due to the acoustic displacement field. However, the theoretical representation of the BLS spectra was qualitative and the elasticity of the NPs was assumed to be isotropic.

In a second experiment of Au nanodevices consisting of Au nanodisks in a regular separation, the blue frequency shift effect of proximity was reported for the main breathing mode οf the Au nanodisk. However, it was observed only for dissimilar (heterodimer) nanodisks, as the up-shifted mode was inactive by symmetry in the case of homodimers.^20^ In the former, the interactions mediated by the substrate even up to 50 nm apart led to a blue shift, which was not captured by FEM simulations.^21^ In spite of the sizeable nanodisks (∼200 nm), the lack of explanation was ascribed to the failure of elasticity theory and suggested that *“more experiments and modeling are, however, needed to further investigate this observed phenomenon″.* A rationalization of the nanodisks interactions was provided based on a phenomenological model of two coupled oscillators.

The strong amplification of the BLS signal near surface plasmon resonance allowed the detection of interparticle interaction effects on the acoustic vibrations of metallic NPs mediated by either plasmon–phonon (optomechanical) coupling or the substrate. In the former case, both the induced quasi-translation mode and the single characteristic fundamental mode are sensitive to the interparticle separation and the environment, the latter albeit to a different extent. Single NP probing is, however, possible to submicron sizes, but its extension to nanometer dimensions, relevant to many applications, is inevitably bound to ensemble experiments. In spite of inherent orientation averaging, exploitation of size scaling, collective effects, and control of the interparticle separation (via ligand grafting) can be better realized at the nanoscale. In this paper, we utilized BLS to record the vibration spectrum of Ag/PVP and polymer-grafted Ag NPs with a diameter between 5.5 and 22 nm. Due to the shift of the Ag extinction spectrum to a lower wavelength compared to Au NPs, both quasi-translation and quadrupolar modes can be probed using a single laser light at 532 nm. We presented thorough theoretical 3D calculations including optomechanical (OM) phonon–plasmon-based coupling to quantify the intensity of the quasi-translation (now termed “rattling”) mode in Ag NPs and hence the BLS activity of the vibration modes of Ag polymer nanocomposites. Since the contribution of the rattling mode depends on the interparticle separation, which in the case of two component polymer nanocomposites is ill-defined, we have employed polymer-grafted Ag NPs which exhibit glassy behavior or liquidlike order with the average distance controlled by the polymer molecular weight and grafting.

## Methods

### Materials

Poly(vinylpyrrolidone) (PVP) and poly(vinyl alcohol) (PVA) with molecular weights of 29 and 27 kDa, respectively, were purchased from Sigma-Aldrich, while PS-SH (2.6 and 18.9 kDa) and PIB-SH (2.6 kDa) were custom-made.^23,24^ Ag NPs with three different diameters in two polymer matrices, PVP and PVA, and two polymer-grafted Ag samples (PIB: poly(isobutylene) and PS: poly(styrene)) were used in the present study. Ag NPs for the Ag-PVP and Ag-PVA nanocomposites were synthesized according to the procedure reported elsewhere.^22^ An aqueous solution of 100 mL of distilled water, containing 0.129 g sodium citrate and 0.017 g tannic acid was added to a 250 mL glass flask equipped with a reflux condenser. The flask was under vigorous stirring with a magnetic bar and placed in a heating bath filled with ethylene glycol at 110 °C. When the solution boiled, 1 mL of silver nitrate aqueous solution (25 mM) was injected. The solution color immediately changed to yellow. After the synthesis, Ag NPs were purified by centrifugation in order to remove the excessive tannic acid and redispersed in distilled water. To prepare Ag-PVP and Ag-PVA composites, Ag NPs obtained by centrifugation of Ag colloidal dispersion (10 mg/mL, 1 mL) were introduced into PVP or PVA ethanol solution (10 mg/mL, 1 mL). The synthesis and characterization of two PIB-grafted Ag NP samples with different Ag compositions (2.3 vol %, 15 vol %) and the same PIB molecular weight (2.6 kDa) and two PS-grafted Ag NP samples with PS molecular weight 2.6 kDa (9.5 vol %) and 18.9 kDa (1.4 vol %) were previously reported.^23,24^

**Figure 1:**
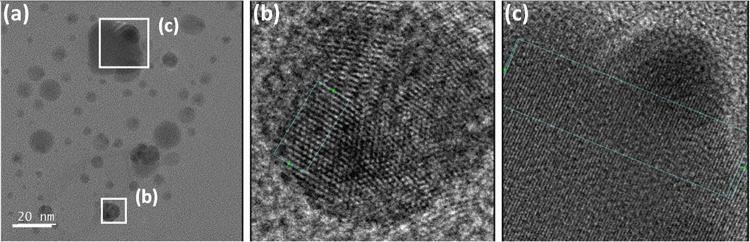
(a) HRTEM image of Ag (14) NP films. (b) Fourier-filtered and magnified image of the rectangular area (b) in (a). (c) Fourier-filtered and magnified image of the rectangular area (c) in (a).

For the extinction UV–vis spectra and the BLS experiments, a few drops of the sample dispersion (Ag-PVP and Ag-PVA in ethanol, Ag-PS and Ag-PIB in toluene) were deposited on cleaned glass substrates. The glass substrates were cleaned by wiping the surface with ethanol before use. Then, the samples were left overnight at room temperature to evaporate all dispersion medium and form a film. In addition, silicon nitride (SiN) substrates were also used to avoid interrupting peaks from glass substrates. SiN substrates (3 mm × 3 mm, 50 nm thick SiN window in 10 mm × 10 mm, 200 um Si frame, model: NX10300A) were purchased from Norcada, Canada. No additional cleaning was needed before use. The Ag-PS films for UV–vis and BLS characterization were prepared by spin-coating. Dispersion of Ag-PS in toluene solution (15 wt %) was deposited on glass substrates. The film on glass substrates was obtained by drying for 10 h in a vacuum oven at 100 °C to evaporate toluene. The sizes of the Ag NPs as determined by transmission electron microscopy (TEM) are listed in Table 1. For each Ag NP sample, at least 100 particles were randomly chosen to yield their average size. The high-resolution TEM (HRTEM) images of the Ag (14) NPs in Figure 1 revealed the crystalline information. The distinct and uniform lattice fringes with d-spacing *d* = 0.24 nm corresponds to a single crystal of (111) atomic planes of silver.^25^

**Table 1:** Diameter of the Ag NPs Dispersed in PVA, PVP, and in Two Polymer-Grafted (PS, PIB) Ag Films

system	Ag NPs dispersed in polymer			polymer-grafted Ag NPs			
material	Ag (14)-PVP29k	Ag (14)-PVA27k	Ag (29)-PVP29k	Ag (22)-PIB3k, 20% Ag	Ag (22)-PIB3k, 65% Ag	Ag (6)-PS3k	Ag (6)-PS 18k
Ag diameter (nm)	14.1 ± 1.6	14.1 ± 1.6	29.4 ± 9.0	22.6 ± 3.3	21.6 ± 3.3	5.5 ± 0.7	5.8 ± 0.9

### Brillouin Light Spectroscopy

BLS is an optical technique based on the scattering of light by thermally excited hypersonic (GHz) phonons that exist in all materials at temperatures higher than 0 K. For an isotropic transparent material, a single acoustic phonon at frequency ω = 2π*f* is probed at a given wave vector (***q***) selected by the scattering geometry. Using energy and momentum conservation laws, ω = *c q*, with *c* being the sound velocity (longitudinal or transverse) in the medium, appears in the spectrum of the scattered light analyzed by a six-pass tandem-Fabry-Perot interferometer.^26^ For localized phonons, as in the present case, the particle resonance frequencies are *q*-independent as revealed by recording the BLS spectra at two different magnitudes of ***q***. In the present work, this was realized by using two distinct scattering geometries, transmission and backscattering. The magnitudes of ***q*** are, respectively, *q*_trans_ = (4π/λ) sin α and *q*_bs_ = 4π*n*/λ, where λ (=532 nm) is the laser wavelength in vacuum, α the laser incident angle, and *n* the material refractive index.^26^ It is worth noting that *q*_trans_ is independent of the sample’s refractive index, which makes this geometry particularly useful for materials with unknown refractive indices.

### Theoretical Modeling

Mechanical eigenmodes of a 14 nm diameter spherical Ag NP embedded in the PVP matrix are determined using full 3D simulations based on the finite element method. The configuration model is sketched in Figure S1, where the Ag NP lies in the center of a large sphere of the PVP material constituting the matrix. The latter is terminated by perfectly matched layers (PMLs) in order to prevent undesirable reflections perturbing the system. An oscillating uniaxial load is applied on one face of the NP for the excitation and then the acoustic resonances are probed following the calculation of the averaged displacement norm, , where *V*_NP_ is the NP volume and *u*, *v*, and *w* are the three components of the displacement field. Adopted elastic parameters for PVP are, *C*_L_ = 2989 m/s, *C*_T_ = 1443 m/s, and ρ_PVP_ = 1200 kg/m^3^, where *C*_L_ and *C*_T_ are the longitudinal and transverse sound velocity, respectively. As for the elastic properties of Ag NPs, an anisotropic elasticity tensor has been used, as implied by the crystalline structure revealed by the HRTEM images (Figure 1). The elastic constants are: *C*_11_ = 123.99 GPa, *C*_12_ = 93.67 GPa, and *C*_44_ = 46.12 GPa.^27^ A quantitative agreement with the experimental fundamental *E*_g_ mode was found without the necessity of adjusting these Ag elasticity parameters.

## Results and Discussion

### Αg Polymer Nanocomposite Films

**Figure 2:**
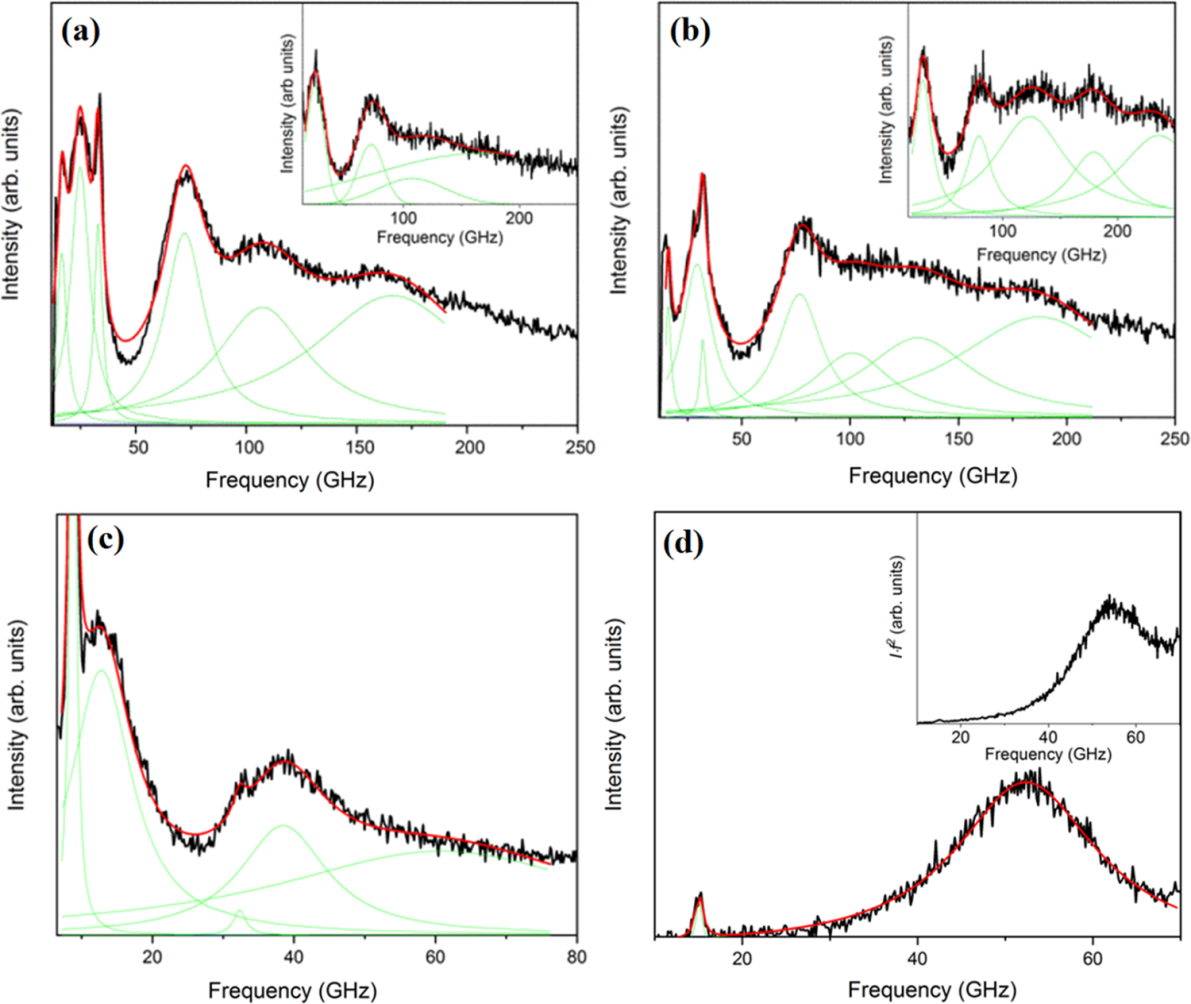
BLS (anti-Stokes) spectra of polymer nanocomposites (a) Ag (14)/PVP, (b) Ag (14)/PVA, (c) Ag (29)/PVP, and Ag (22)/PVP on glass (main panels) and the SiN substrate (insets to (a) and (b)) with Ag diameter *D* = 14.1 nm (a,b), *D* = 29.4 nm (c), and *D* = 21.6 nm (d). The inset to (d) is the reduced spectrum, intensity × frequency square, of the spectrum in the main figure. The representation of the spectra by Lorentzian lines (green lines) is indicated by the solid red lines (see text).

Figure 2 displays experimental BLS spectra (anti-Stokes side) for three Ag/PVP films with different Ag diameters *d* (panels a, c, and d) and two polymer matrices, PVP (a) and PVA (b), along with their representation (red line) by a sum of Lorentzian line shapes (green lines). The films in the main panels of Figure 2 are glass-supported, whereas the spectra in the insets to (a) and (b) refer to Ag (14)/PVP and Ag (14)/PVA films on SiN; the numbers in parentheses indicate the Ag diameter. The glass (SiN) substrate is also evident from the presence (absence) of the longitudinal acoustic phonon (sharp peaks) in the glass. Based on the extinction spectra (Figure S2) with maximum absorption at ∼428 nm, the BLS spectra recorded at 532 nm are off-resonance with respect to individual Au NPs. Several findings emerge from Figure 2 and the corresponding Figures S1 and S2: (1) The main feature is the low frequency peak (24 GHz) while the first high frequency peak (72 GHz in Figure 2a) demonstrates the nanoparticle’s own signature, i.e., the analogue of the quadrupolar mode in the case of elastically isotropic Ag NPs. The particle size polydispersity is reflected in the linewidth of the quadrupolar mode and the line broadening increases with the decreasing NP diameter (Figure 2a,c).^28^ However, this is not the subject of this work. (2) For Ag (22)/PVP (Figure 2d), the low frequency mode is not discernible and only the high frequency mode is present. (3) The polymer matrix composition addressed in the case of Ag/PVP affects the intensity but not the shape and the frequencies of the modes (Figure S3a). The substrate has also negligible, within the experimental error, effect on the BLS spectra of Ag/PVP1 (Figure S3b). (4) The polymer matrix (PVP, PVA) has a discernible effect on both frequencies being blue-shifted in PVA. (Figure 2a,b and Figure S3c). The frequencies of the different modes from the experimental spectra are listed in Table 2. (5) An almost “dark” valley between the two localized modes is apparent in all samples, apart from Ag (22)/PVP, which remarkably displays only the high frequency mode. This feature was absent in the previous BLS studies.^14,17,18^

**Table 2:** Frequencies of the Low and High Frequency Modes in Ag/PVP and Ag/PVA on the Glass Substrate

sample	*f*_1_ [GHz], *f*_1_D [m/s]	*f*_2_ [GHz], *f*_2_D [m/s]	*f*_3_ [GHz]	*f*_4_ [GHz]	*f*_5_ [GHz]
Ag (14)/PVP	24 ± 1, 340 ± 15	74 ± 2, 1040 ± 20	107 ± 22	166 ± 38	
Ag (14)/PVA	29.2 ± 0.5, 410 ± 10	79 ± 2, 1110 ± 20	101 ± 19	131 ± 27	187 ± 40
Ag (22)/PVP		52.4 ± 11, 130 ± 10			
Ag (29)/PVP	12.8 ± 0.7, 380 ± 20	38.5 ± 0.8, 1130 ± 20			

**Figure 3:**
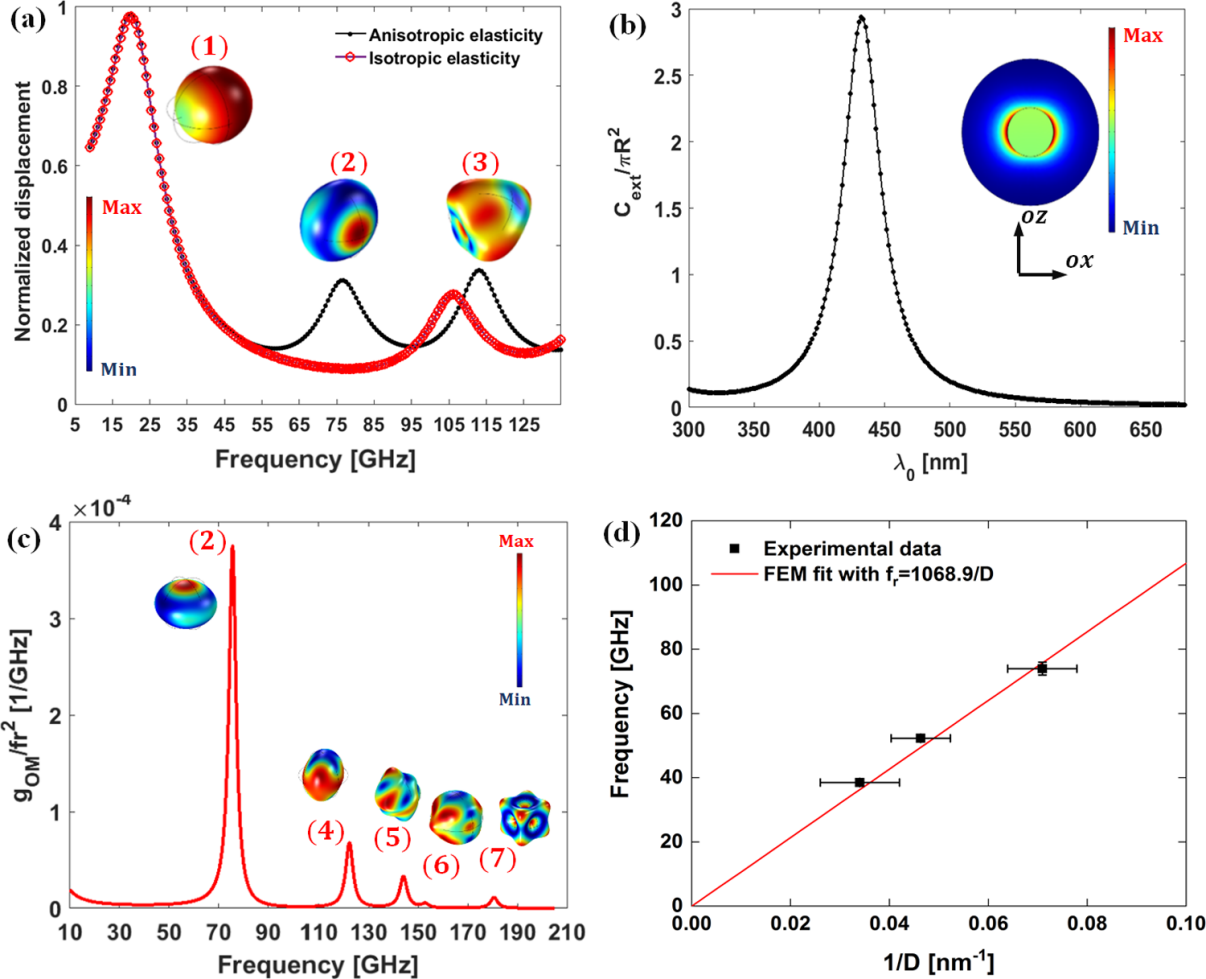
(a) Computed displacement spectrum for single Ag (14) in PVP in the case of anisotropic elasticity (black curve with black dots) and isotropic elasticity (red symbols). Inset: The displacement map norm within the Ag NP associated with modes referred to as (1), (2), and (3) at ≈ 20, 76.6, and 113 GHz, respectively. (b) Simulated extinction cross-section of Ag (14)/PVP. Inset: The scattered E-field norm map depicted over a cross-section of the NP in the *ozx*-cut plane at λ_0_ = 532 nm. (c) Simulated unpolarized OM coefficients versus the frequency for a Ag (14) NP placed in the PVP matrix (Figure S1a) convoluted with a Lorentzian curve having a full width at half maximum set to Δ*f* = 1.5 GHz, and then divided by the square of frequency so to mimic the BLS spectrum of thermally excited phonons. Inset: The displacement map norm within NPs associated with the *E*_g_ mode (2) and higher frequency modes (4) to (7). (d) Frequency of the fundamental *E*_g_ (red line) computed for single Ag NP in the PVP matrix (red line slope 1069 m/s) as a function of the Ag diameter *D*.

Prior to the comparison with the theoretical predictions, the issue of the Ag elasticity should be first clarified. In all reports so far, but one, an isotropic elasticity was assumed using an angular average of the elastic tensor of the crystalline Ag. Since the latter is clearly revealed by the HRTEM images of Figure 1, we first compared the experimental frequency of the high frequency mode, which is a signature of the Ag NPs, with the theoretical estimates obtained assuming either isotropic or anisotropic Ag elasticity. In Figure 3a, we show the computed displacement spectrum associated with the anisotropic elasticity tensor (black curve with black doted markers) and the spectrum corresponding to isotropic elasticity (circular red markers); in the latter case, we use the orientation averaged values, *C*_L_ = 3747 m/s and *C*_T_ = 1740 m/s.^29^ Both displacement spectra are normalized with respect to the displacement measured in plain PVP in the absence of the Ag monomer. In the case of isotropic elasticity, the five times degenerate quadrupolar (*n* = 1, *l* = 2) mode appears at ∼106 GHz (Figure 3a, black symbols), which largely exceeds the experimental value (74 GHz) for Ag (14)/PVP (Figure 2a). Invoking anisotropic elasticity, the high frequency mode (2) in Figure 3a (black symbols) corresponds to the two times degenerate fundamental eigenmode of the NP with irreducible representation *E*_g_ at ≈76.6 GHz in close proximity with the experimental value. Based on this agreement, we adopt the anisotropic elasticity approach to model mechanical properties of the Ag NPs in our study. In the inset to Figure 3a, we present the excited eigenmode shapes (1), (2), and (3) of the single Ag NPs. Mode (1) is the so-called rattling (or constrained translational) mode arising because of the elastic force of the PVP matrix, mode (2) is the *E*_g_–fundamental mode mentioned above and mode (3) is a higher frequency spheroidal type mode found at about 113 GHz.

In the case of single Ag NP vibrations, the theory predicts an additional low frequency quasi-translational mode (1) at ∼20 GHz, which is robust to the Ag elasticity (Figure 3a). In order to examine the BLS activity of the predicted (1) and (2) modes, we have considered the OM coupling between the excited optical mode and elastic vibrations of the Ag NPs at the operating laser wavelength, λ_0_ = 532 nm. The extinction cross-section of the single Ag NP computed using the Lorentz–Drude model (Section S4) is shown in Figure 3b. The localized surface plasmon (LSP) resonance occurs at λ*_r_* ≈ 430 nm and compares well with the experimental maximum absorption at ∼428 nm (Figure S2) with no adjustable parameters. The associated scattered *E* field map norm is depicted on a cross-section of the NP (*ozx*) in the inset to Figure 3b. Albeit the operating λ_0_ does not match λ*_r_*, the LSP mode can yet be excited but off resonance meaning that the OM coupling can still take place. It originates from two physical mechanisms, the so-called moving interface (MI) and the photoelastic (PE) effect.^30−32^ In the case of MI, a local change of the electric permittivity is induced near the surface, whereas the PE effect operates in the bulk of the material. The expressions for the OM coefficients, *g*_MI_ and *g*_PE_, are obtained based on a first-order perturbation theory^30,33^ and from the knowledge of the acoustic and optical fields in the NP (Section S4). These expressions are commonly used to evaluate the phonon–photon coupling in optomechanic cavities where acoustic and optical waves are confined simultaneously.^34^ Hence, we simulated the OM coefficients, *g*_OM_, to identify the BLS active vibration modes which strongly couple with the plasmon. Then, the OM coupling rate defined as *g*_OM_ = *g*_MI_ + *g*_PE_ is shown in Figure S4.

The rattling mode (1) and the high frequency mode (3) observed in Figure 3 both present negligible OM values compared with other modes. The BLS spectrum is represented by *g*_OM_/*f*^2^ in Figure 3c where the factor *f*^2^ in the denominator accounts for the phonon thermal population. Modes (1) and (3) are inactive in the BLS experiment due to the selection rules dictated by modes symmetry^14^ and consequently negligibly low *g*_OM_ in Figure S4. On the other hand, modes (2), (4)–(7) present important *g*_OM2_ ∼ 3.7 GHz > *g*_OM4_ ∼ 1.76 GHz > *g*_OM5_ ∼ 1.18 GHz > *g*_OM6_ ∼ 0.17 GHz > *g*_OM7_ ∼ 0.63 GHz. Here again, the difference in these observed values relates to the overlap of the mechanical modes with the plasmon and the OM coupling magnitude.

In the case of a single Ag NP, the computed frequency of the fundamental *E*_g_ conforms to the expected size scaling, *f*_2_ (GHz) = 1069/*D* in the PVP matrix (the red solid in Figure 3d); in air *f*_2_(GHz) = 1033/*D*. Hence, the surrounding solid medium can marginally (∼3.5%) impact the fundamental *E*_g_ frequency. On the experimental side, the *E*_g_ frequency of the Ag NPs in PVP for three different diameters (Table 1, Figure 3d), compares well with the theoretical prediction for the single crystalline Ag NP in PVP. However, the low frequency mode is absent in the theoretical calculations (Figure 3c) in the case of single Ag NP, in agreement with the literature reports.^14,17^ Instead, a dimer of Ag NPs was invoked to account for its presence in the BLS spectra.

**Figure 4:**
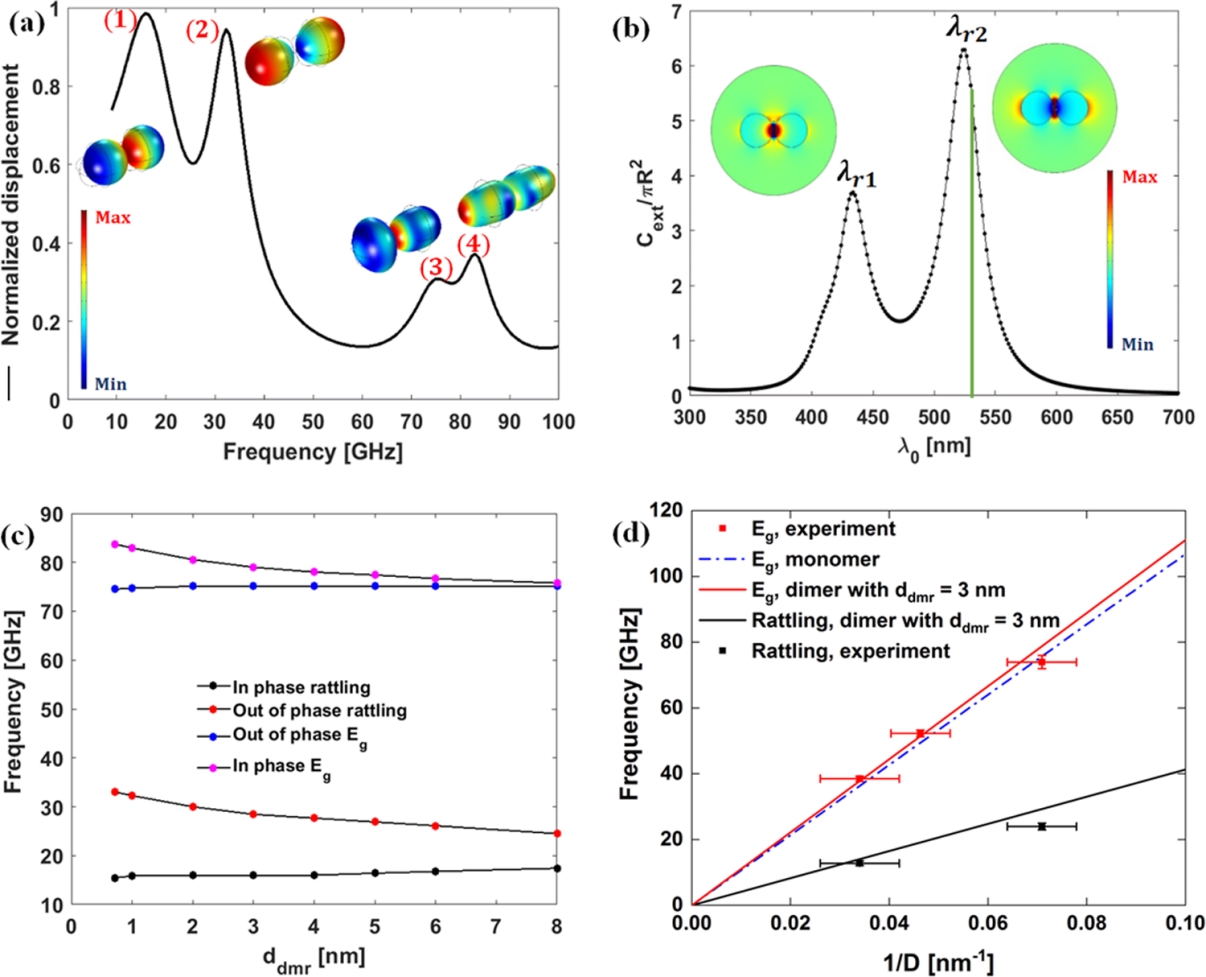
(a) Computed displacement spectrum corresponding to the dimer geometry (Figure S1b) of 14.1 nm diameter Ag in the PVP matrix for a separation, *d*_dmr_*=* 1 nm. Inset: The absolute displacement field map norm within Ag NPs associated with split modes referred to as (1), (2), (3), and (4). (b) Normalized extinction cross-section of the dimer and the *x*–component of the scattered *E⃗*–field with resonance wavelengths λ_*r*1_ ≈ 432 nm and λ_*r*2_ ≈ 525 nm.The vertical green line at 532 nm denotes the wavelength of the excitation laser light. (c) Frequency of the rattling mode (out-of-phase red, in-phase black symbols) and the *E*_g_ mode (out-of-phase blue, in-phase pink symbols) for a Ag dimer in the PVP matrix as a function of the Ag–Ag separation *d*_dmr_. (d) Frequency of the out-of-phase rattling (black line) and in-phase *E*_g_ (red line) computed for the Ag dimer in the PVP matrix as a function of the Ag diameter *D* for a separation, *d*_dmr_ = 3 nm. The dashed blue line denotes the prediction of the fundamental *E*_g_ for a single Ag in the PVP matrix. The experimental frequency of the rattling and *E*_g_ modes for Ag with three different Ag diameters in PVP is denoted by black and red squares, respectively.

On the theoretical side, the vibration spectrum of the Ag dimer in the PVP matrix is qualitatively and quantitatively different from that of the Ag unimer, consistently assuming anisotropic elasticity. For the Ag (14) dimer at edge–edge separation *d*_dmr_, a harmonic load was applied on one of the Ag NP surfaces along the dimer axis to probe the mechanical modes (Figure S1b). In view of the presence of various types of multimers, we note that the dimer case represents the simplest structure to simulate coupling effects. The averaged displacement norm computed within NPs and normalized with respect to the displacement in plain PVP in the absence of the dimer is recorded within the Ag NP volume. The computed displacement spectrum for *d*_dmr_ = 1 nm presented in Figure 4a reveals a frequency split for both the low frequency mode (rattling) and the fundamental *E*_g_ mode denoted as (1) to (4). Based on the displacement field norm maps within the NPs depicted in the inset to Figure 4a, the modes (1) and (4) correspond to in-phase vibration modes, whereas for modes (2) and (3), the dimer vibrates out-of-phase. The split is particularly strong for the low frequency vibration as modes (1) and (2) are nearly Δ*f* ∼ 16 GHz apart while Δ*f* ∼ 8 GHz for modes (3) and (4).

Since the rattling mode relates to the restoring elastic force of the PVP matrix, the effect of coupling between the NPs (forming the dimer) mediated by the polymer on the mode frequencies is much stronger than that for the fundamental *E*_g_ mode due to its stronger localization within the NPs. Note that there is no rattling mode in air due to the absence of coupling between the NP spheres (unless they are in contact). Figure 4b displays the normalized (with respect to NP cross-sections) extinction spectrum for the dimer configuration (Figure S1b) for *d*_dmr_ = 1 nm and the incident electric field polarized along the dimer axis. In contrast to the single peak extinction (λ*_r_* ≈ 430 nm) of the single Ag NP in PVP, two distinct plasmon resonances, at λ_*r*1_ ≈ 432 nm and λ_*r*2_ ≈ 525 nm are active in the case of the Ag dimer in PVP. Based on the *x*–component of the scattered *E*–field at λ_*r*1_, λ_*r*2_, shown in Figure 4b, the excited modes are surface plasmon resonances (LSP) being strongly localized between the Ag NPs. The experimental UV–vis absorption spectrum (Figure S2) displays a very broad peak at λ ≈428 nm extending up to about 600 nm like the simulated spectrum in Figure 4b. However, unlike the latter (computed for a single Ag dimer) the second peak is not discernible indicating a moderate population of dimers in the Ag/PVP sample. Noticeably, the second high wavelength peak is present in the experimental UV–vis absorption spectrum of Ag in PVA (Figure S2).

Simulations of the OM coupling between mechanical modes (Figure S5a) of the dimer and the surface plasmon excited at λ_0_ = 532 nm (closer to λ_*r*2_,vertical line in Figure 4b) distinguish the contribution of the four vibration modes of Figure 4a to the experimental BLS spectra. Accordingly, only the out-of-phase rattling mode (2) and the in-phase fundamental *E*_g_ mode (4) are the BLS active modes, as both strongly deform the space between the NPs where LSP are localized. Consequently, the overlap rate between the LSP and mechanical modes (2) and (4) is high resulting in a strong interaction. This finding is consistent with earlier reports showing a similar trend.^14,17^ Mode (4) possesses more than twice higher OM rate than mode (2) in the Ag dimer at *d*_dmr_ = 1 nm (Figure S5a). However, the intensity of the low frequency mode (2) in the spectrum *g*_OM_/*f*^2^ is higher than that for mode (4) because of the stronger mode (2) thermal modulation (Figure S5b for *d*_dmr_ = 1 nm and *d*_dmr_ = 3 nm). The intensity contribution of the low frequency mode (2) is very sensitive to the separation: For mode (2), the OM coupling decreases nearly by half with increasing *d*_dmr_ from 1 to 3 nm and essentially vanishes for interparticle separation more than about 10 nm (Figure S6). For mode (4), the OM coupling and hence its intensity is rather robust to the *d*_dmr_variation assuming *g*_OM_ ∼7.5 GHz, which is twice the value for a single Ag NP (red solid line in Figure S6). The OM coupling between the SPR plasmon and mode (4) is therefore governed by individual Ag NPs also meaning that all dimers contribute equally to the particular intensity in the BLS spectrum. In this context, we should mention that we have considered only homodimers. On the contrary, in the case of Au nanodisks, the blue frequency shift effect of proximity was observed only for dissimilar (heterodimer) nanodisks.^21^

The computed frequencies for the Ag dimer for the two BLS active modes are shown as a function of *d*_dmr_ in Figure 4c. Both modes undergo a blue frequency shift for particle separation, *d*_dmr_ < 8 nm. Note that the frequencies of the BLS inactive in-phase rattling and out-of-phase *E*_g_ modes are insensitive to the Ag separation in the dimers as their coupling is insufficient to induce sufficient deformation for a discernible blue shift. The experimental frequencies of the BLS active modes of Ag with three diameters in PVP is shown in Figure 4d along the computed dependence of the *E*_g_ frequency for single Ag (blue line) and *E*_g_ (red) and rattling (black line) frequencies for the Ag dimer at *d*_dmr_ = 3 nm. The corresponding theoretical slopes (*fD*) amount to 1111 m/s (red line) and 413 m/s (black line). As already mentioned, the experimental *E*_g_ is close to the theoretical prediction in the case of single Ag NPs in PVP (see also Figure 3d). Since BLS probes an ensemble of Ag NPs at different mutual separations and the frequency of *E*_g_ ranges theoretically between 76 and 83 GHz (for *D* = 14.1 nm; Figure 4c) with all contributing equally to the spectrum (Figure S6), the latter finding suggests high population of single (*d*_dmr_ > 5 nm) Ag NPs. This presumption is supported by the comparable intensities for the rattling and *E*_g_ modes (Figure 2a), which favors relatively large intraparticle distances (Figure S6b). For the two other Ag/PVP samples with larger *D*, the average *d*_dmr_ (=3 nm in Figure 4d) slightly decreases. Notably in the case of Ag(22), no rattling mode is discernible in the BLS spectrum of Figure 2d suggesting the absence of Ag dimers with *d*_dmr_ < 10 nm, as confirmed by the SEM image (Figure S7). For the rattling mode, the slope is much lower and depends on the matrix elasticity. The comparison between experimental (Table 2) and theoretical (Figure 4d) values of the rattling frequency for Ag (14)/PVP and Ag (29)/PVP samples suggests the presence of physical dimers at a moderate proximity (*d*_dmr_ ∼ 3–5 nm). We should note that the obtained agreement was obtained with no adjustable (matrix and Ag elasticity) parameters.

In the comparison of the experimental frequencies, we were aware of the possible experimental uncertainties: (i) Size polydispersity of the Ag NPs (about 10%) can lead up to about 10% broadening in the case of single Ag NPs; (ii) the dimer contribution results in a blue shift of about 9 and 17 GHz for the high frequency modes in the case of Ag (14 nm) decreasing with separation *d*_dmr_ (Figure 4c, Figure S5b); (iii) size polydispersity of each sphere has a small effect on the rattling frequency and a size asymmetry of 1.15 causes a small (∼3%) red shift in the heterodimer compared to the homodimer; and (iv) in the frequency range of *E*_g_ modes, the dimer contributes to the BLS spectrum almost equally as two single NPs (Figure S6 at 532 nm) that renders dimer/monomer population but not their proximity (Figure 4b) irrelevant. In the case of the same Ag NPs in the PVA matrix, the size polydispersity is not an issue and the blue shift of both modes with respect to the PVP matrix (Table 2, Figure S3) could, in principle, be attributed to the harder PVA matrix (*C*_L_ = 3400 m/s, *C*_t_ = 1470 m/s, ρ *=* 1069 kg/m^3^) and/or different NP proximity (Figure S8). Both parameters have stronger impact on the rattling than the *E*_g_ frequency, and in fact, the experimental value in PVA (Table 2) can be well represented (30 GHz) for Ag dimers at *d*_dmr_ ∼ 3 nm (Figure S8). On the other hand, the *E*_g_ frequency for single Ag should be virtually the same in both PVP and PVA matrices so that the higher (∼7%) value in PVA is rather ascribed to the Ag dimer contribution. Based on the UV–vis spectra (Figure S2a), the dimer population in close proximity (Figure 4b) is higher in PVA than in PVP.

One unexpected feature of the experimental vibration spectra in Figure 2a–c was the observation of a dip valley in the intensity between the rattling and *E*_g_ modes. Note that the sum of two broad Lorentzians, for the rattling and *E*_g_ modes, plus the contribution of high order modes overestimates the minimum intensity at the dip thus reflecting the frequency separation between the modes. The intensity at this “valley” assumes the baseline at very high frequencies (Figure 2a,b). Physically this would require a Fano interference of at least two different modes, for example, a broad propagating and a narrow localized (resonance) mode.^35^ In particular for plasmonics, Fano resonances in the extinction spectrum can be obtained by destructive interference between two localized dipolar modes.^36,37^ In that case, the valley could represent an antiresonance of the Ag/PVP system, which is always between two resonances of the same system, e.g., the rattling and *E*_g_ vibration modes. However, the elucidation of this new finding requires additional studies in the near future about the contribution to BLS of all the modes in the vicinity of the nonresonant frequency range of the valley.

In summary, the present complete theoretical calculations of the active vibration modes of plasmonic nanoparticles including optomechanical coupling for the mode amplitudes led to an unambiguous assignment of the modes identifying their nature and pertinent parameters. The assumption of anisotropic elasticity of Ag NPs is justified, and matrix elasticity was found to dominate the rattling frequency, whereas the dimer length influences the frequency of both modes. In addition, the population of dimer is crucial for the intensity of the rattling mode. However, both the dimer length scale and population are uncertain parameters not accessible independently. For this reason, we examine next the case of polymer-grafted Ag using fluid (PIB) and glassy (PS) grafts with controlled constant average separation between Ag NPs in their assembled structure. Polymer-grafted nanoparticle (GNP) assemblies display unique optical and phononic properties^38−40^ compared to polymer nanocomposites due to their anisotropic density profile.^41^

### Single Component Polymer-Grafted Nanocomposites

**Figure 5:**
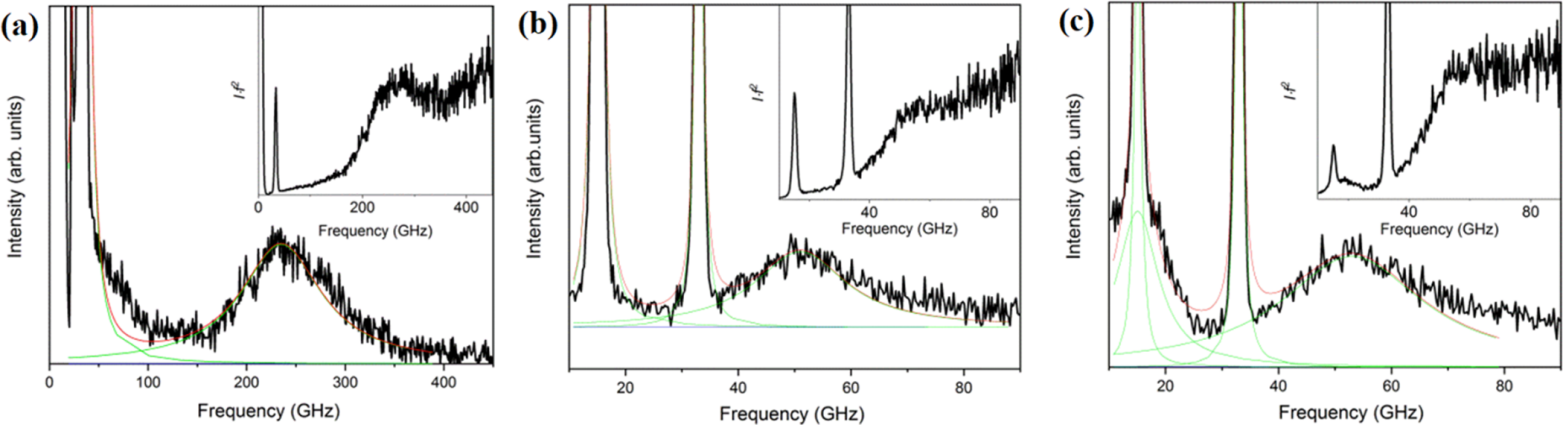
Anti-Stokes side of the BLS vibrational spectra of three polymer-grafted Ag films: (a) Ag@PS3k, (b) Ag@PIB3k with 20% metal, and (c) Ag@PIB3k with 65% metal (see Table 3). The insets show the reduced spectra of the main figures and the sharp peaks relate to the glass phonons at 90° (∼16 GHz) and artificial back scattering (∼32 GHz).

The two polymer grafts, PS and PIB, were considered on the account of their glass transition temperature being either well below (PIB) or above (PS) the ambient temperature. Moreover, the average distance between neighboring Ag NPs was controlled by changing the polymer molecular weight (Ag@PS3k andAg@PS18k)^42^ or the grafting density (Ag@PIB3k with20%or 65% metal).^23^ The edge-to-edge interparticle separation in Ag (5.8)@PS18k is sufficiently large (*d*_dmr_ = 16 ± 1 nm)^42^ so single Ag NP calculations (Figure 3) can be safely assumed. Expectedly, the BLS spectrum displays only the fundamental *E*_g_ mode at 220 GHz (Table 3) since there is no evidence of a rattling mode. For comparison, the BLS spectrum of Ag (5.5)@PS3k (Figure 5a) also displays a single *E*_g_ mode at slightly higher frequency (230 GHz) due the correspondingly smaller diameter (Table 1). Again, there is no evidence of a rattling mode at low frequencies even after correction of the thermal phonon population (inset to Figure 5a). The absence of the rattling mode in the latter GNP with much higher Ag content implies that even at *d*_dmr_ = 4.8 ± 1.3 nm ∼ 5 nm, Ag@PS GNPs rattling mode is not discernible. Using bulk PS sound velocities, *C*_L_ = 2380 m/s and *C*_T_ = 1150 m/s, and density, ρ_PS_ = 1040 kg/m^3^, the computed frequency (≈191 GHz) of the *E*_g_ mode is about 15% lower than the experimental value (Table 3) and about 24% higher than that in air (≈178 GHz). Using bulk PS elasticity and one Ag@PS dimer 5 nm apart, the simulations showed that the rattling mode mediated by the polymer is weak compared to the PVP and PVA matrix (Figure 4) probably due to the softer PS. Its frequency is therefore estimated at ≈48 GHz being much lower than anticipated (≈73 GHz) for the small (5.5 nm) Ag diameter in PVP (Figure 4d). Based on this theoretical prediction, the absence of the rattling mode in the experimental spectra of Figure 5a is conceivable.

**Table 3:** Frequencies of the Low and High Frequency Mode Films in Polymer-Grafted Ag NP Films on the Glass Substrate

sample	Ag (22)@PIB3k (20% metal)^a^	Ag (22)@PIB3k (65% metal)^a^	Ag (5.5)@PS3k^b^	Ag (5.8)@PS18k^b^
rattling f_1_ [GHz]), f_1_D [m/s]		19 ± 2, 410 ± 20		
*E*_g_ f_2_ [GHz], f_2_D [m/s]	51 ± 2, 1130 ± 20	53 ± 1, 1130 ± 20	230 ± 4, 1265 ± 20	220 ± 3, 1275 ± 20

a Ref (23).

b Ref (24, 42).

Assuming for the crystalline Ag NP elastic anisotropy, the next attempt is to adjust the elasticity of the PS grafts. It is accepted that the structure of GNP assemblies comprises two different length scales, *h*_dry_ with the higher density region next to the Ag core than the outer region and *h*_wet_, where chain interpenetration of PS grafts from adjacent cores occurs, Figure S9a.^41^ Accordingly, we have attempted to adjust the elasticity of the shell of *h*_dry_ between 2 and 3 nm and *d*_wet_ between 6 and 5 nm; these numbers are estimated based on the end-to-end distance of PS with 181 monomers and *d*_dmr_ = 16 ± 1 nm for Ag@PS18k.^41^ To simulate the experimental frequency of the *E*_g_ mode, we first adjust the PS elasticity in the dry region (*h*_dry_ = 2 *nm*), while maintaining the interpenetration region (*h*_wet_ = 6 nm) with PS elasticity; only the transverse sound velocity *C*_T_ has a notable effect as the frequency is insensitive to the increase of *C*_L_. The approach does not capture the *E*_g_ frequency (220 GHz) of the Ag@PS18k system. Then, we analyzed the effect of hardening for both the outer interpenetration and dry region elasticity (the same elasticity is applied in both regions). This particular simulation (not shown) indicated that an extremely high *C*_T_ (∼5000 m/s) would be required to capture the experimental frequency of the *E*_g_ mode. This corresponds to about 20 times higher Young’s modulus, *E* (=2*G*(1 + *v*)) for the grafted PS which is physically unrealistic. Thus, a reasonable increase in the PS elasticity is insufficient to approximate the experimental *E*_g_ frequency implying the necessity of an additional constraint on the Ag NP motion. One possibility to impose a rigid condition is a vanishing velocity, i.e., *u⃗* = 0⃗ in the system but the position is not obvious. In order to examine the effect of this constraint, we applied it at the outer boundary of the double-layer-core model of Figure S9a. The computed *E*_g_ frequency versus *C*_T_ (Figure S9b) at a constant *C*_L_ and in the case that only the dry region is hardened also requires an unacceptable high *C*_T_ (∼2800 m/s) to capture the experimental *E*_g_. The latter can be approximated if both (dry and wet) layers are allowed to become harder with *C*_T_ in the range (1450–1650) m/s depending on the PS thickness, ensuring a slightly harder dry region (Figure S9c). One can claim that other possibilities and assumptions can also be envisaged, for example, constrain the tangential motion of the NP at its own surface by the PS grafts or consider the dry region highly anisotropic with respect to the normal and tangential directions.^43^ This could be the object of a future work, perhaps in connection to other experimental information for nanocomposites with different sizes of the NPs or theoretical modeling of the interfacial layer based on the structural information on the GNPs. The possibility that the Ag NPs have a much smaller (∼20%) diameter is excluded^42^ and is not supported by the scale of the *E*_g_ frequency by their experimental diameter (Table 3).

For the two Ag@PIB samples with 20 wt % (2.3 vol %, *D* = 22.5 nm) and 65 wt % (15 vol %, *D* = 21.6 nm) Ag composition, the estimated separation amounts to *d*_dmr_ ∼ 5 and ∼14 nm, respectively.^23^ Prior to the discussion of the vibration spectrum, Ag@PIB GNPs display two distinct differences compared to the Ag@PS GNPs: In the bulk state, PIB is fluid and the PIB grafts are extremely confined (larger core and small polymer size). The simulations of the single Ag (*D* = 21.6 nm) vibration in the liquid PIB matrix (*C*_L_ = 2590 m/s, *C*_T_ = 0, ρ_PIB_ = 920 kg/m^3^) predict only the fundamental *E*_g_ mode at 45.8 GHz (Figure S10a) which is lower than that in air (47.8 GHz, Figure 3d). More importantly and unexpectedly, the experimental *E*_g_ frequency (53 GHz, Table 3) is found to be significantly higher (by about 15%) than the computed value. The small but discernible difference (∼4%) in the *E*_g_ frequencies in the two Ag@PIB samples is due to the slight disparity in the Ag core diameters. Surprisingly, the experimental *E*_g_ frequency is close to the anticipated value (∼50 GHz, Figure 3d) in the rigid PVP matrix.

To relate this large difference to the polymer rigidity (glass vs liquid), we also examined the same Ag (22) NPs in the PVP matrix (Figure 2d). For the latter, the experimental frequency (52 GHz) of the *E*_g_ mode (Table 2) is in very good agreement with the computed value for a single Ag NP in the PVP matrix as already discussed in the previous section (Figure 3d). However, the unexpected similarity observed between the data in the grafted PIB matrix and the PVP dispersion inevitably suggests a modification of the matrix elasticity at least in the Ag–PIB interface region (Figure S10). Moreover, the experimental BLS spectrum of Ag@PIB (65%) in Figure 5c displays a low frequency mode at ∼19 GHz (Table 3). This finding is also not supported by the simulations of the Ag dimer at *d*_dmr_ ∼ 5 nm in the PIB liquid matrix.

Based on the solid experimental findings, we have performed simulations of the Ag(21.6 nm) dimer in the PIB matrix with constant *C*_L_ (=2590 m/s) and separation *d*_dmr_ = 5 nm and variable *C*_T_ reflecting a solidification of the confined PIB (Figure S10). With an increasing *C*_T_ value a rattling mode evolves at low frequencies and the *E*_g_ mode displays a blue shift toward to the experimental values for both vibration modes in Ag (21.6)@PIB with 65 wt% metal (Figure 5c). The notable rigidity of PIB (*C*_T_ ∼ 1400 m/s) is supported by the optical appearance of these Ag@PIB films as a shiny scratchy solid. The absence of the rattling mode in the experimental spectrum of Figure 5b of Ag (22.5)@PIB with 20 wt% metal is probably related to the lower Ag fraction but mainly to almost twice larger separation of the Ag cores compared to Ag@PIB with 65% metal GNPs (see Figure 4c).

## Conclusions

Out-of-phase rattling and in-phase *E*_g_ vibration modes are active in BLS of Ag spheres in the solid polymer matrix. Theoretically, the former is absent for Ag unimers which applies for homogeneous dispersions, whereas the latter which is the fundamental mode of single Ag NPs is slightly blue-shifted in Ag dimers. The solid polymer matrix (PVP, PVA) causes a small (∼4%) blue shift of the *E*_g_ frequency relatively to the free space, whereas the polymer elasticity strongly increases the rattling frequency because it mediates the mechanical coupling between two Ag NPs. These vibration features quickly decay with the interparticle separation and resemble the distance-dependent plasmon coupling of noble metal NPs leading to surface plasmon resonance shifts and strong localization of the light in the gap regions. These unique plasmon features guided the development of highly sensitive chemical and biological sensors.^44,45^

The assembled polymer-grafted Ag NPs with a controlled constant average interparticle distance and the confinement of the polymer grafts were found to strongly influence the Ag NP vibrations. In the case of glassy (PS) grafts, the significant (∼25%) blue shift of the *E*_g_ frequency cannot be reproduced by the simulations using physically meaningful PS elasticity. Instead, other mechanisms to reduce the Ag NP mobility need to be proposed. Keeping the grafted polymer architecture, the polymer rigidity was lifted in the case of PIB which does no support shear waves in the bulk state. For the fluid polymer matrix, no rattling mode is supported and the *E*_g_ frequency is red-shifted compared to a rigid polymer environment. In contrast to these predictions, both the presence of the rattling mode and the blue shift of the *E*_g_ frequency are quantitatively reproduced assuming a solidification of the highly confined polymer grafts in the interparticle spacing. In addition to the physics of the plasmonic NP vibrations, new polymer behavior can be revealed from the optomechanically enhanced vibration spectrum of the noble metal nanostructure.

## Supporting Information Available

Sketch of the employed 3D model to probe mechanical eigenmodes in spherically shaped Ag unimers and dimers; UV–vis absorption spectra of dried Ag-PVP and Ag-PVA films, as well as PS- and PIB-grafted Ag NP films on glass substrates; normalized to the peak intensity of the high frequency peak BLS spectra of Ag/PVP and Ag/PVA nanocomposites; theoretical modeling of optomechanical coupling; simulated unpolarized optomechanical coefficients versus frequency for a Ag NP placed in the PVP matrix, for *d*_dmr_ = 1 nm and *d*_dmr_ = 3 nm; computed optomechanical coefficients associated with out-of-phase rattling and in-phase *E*_g_ modes; SEM image of the Ag/PVP film; frequency of *E*_g_ in a Ag unimer and the rattling mode in the case of the Ag dimer; sketch of the employed double layer core model and corresponding simulated *E*_g_ frequency versus shear speed of the inner dry region, as well as in case where both dry and wet region shear velocities are simultaneously adjusted; computed eigenmode localization rate versus frequency in a single Ag; and simulated vibration spectrum of Ag/PIB showing a rattling mode to evolve with increasing PIB rigidity, in addition to the *E*_g_ mode (PDF)
