# Supplementary material for: Optomechanic Coupling in Ag Polymer Nanocomposite Films
Source: J Phys Chem C Nanomater Interfaces. 2021 Jun 30;125(27):14854–64. doi: 10.1021/acs.jpcc.1c04549 (PMC8287562; doi:10.1021/acs.jpcc.1c04549)
Supplement: Supplementary file 1 — jp1c04549_si_001.pdf [file jp1c04549_si_001.pdf]

# Supporting Information

**Optomechanic Coupling in Ag Polymer Nanocomposite Films**

Adnane Noual[1], Eunsoo Kang[2], Tanmoy Maji,[3] Manos Gkikas[3], Bahram Djafari-Rouhani[4], George Fytas[2,*]

[1] Université Mohammed Premier, Faculté Pluridisciplinaire Nador, LPMR, BP 717-60 000 Oujda, Morocco.
[2]Max Planck Institute for Polymer Research, Ackermannweg 10, 55128, Mainz, Germany
[3]University of Massachusetts Lowell, Department of Chemistry, Lowell, Massachusetts 01854, USA
[4]Institut d'Électronique, de Microélectronique et de Nanotechnologie (IEMN), UMR-CNRS 8520, Department of Physics, University of Lille, 59655 Villeneuve d'Ascq, France

## S1. Configuration of a Ag unimer and Ag dimer

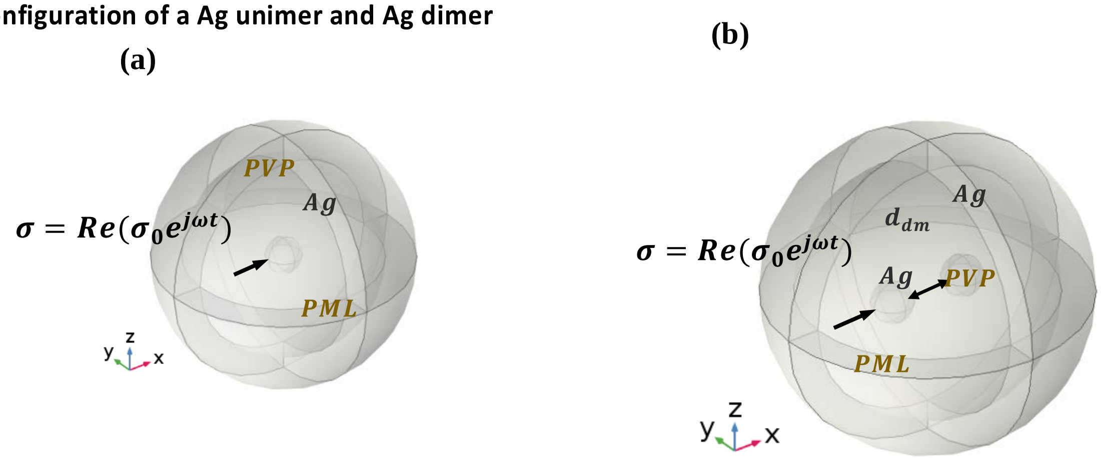


**Figure S1.** Sketch of the employed 3D model to probe Ag (14) NP mechanical eigenmodes in spherically shaped PVP matrix which surrounded by perfectly matched layers (PMLs) for the elimination of undesired reflections. Single Ag NP (a) and dimer at an interparticle separation, $d_{\mathrm{dmr}}$ (b).

## S2. Experimental extinction spectra

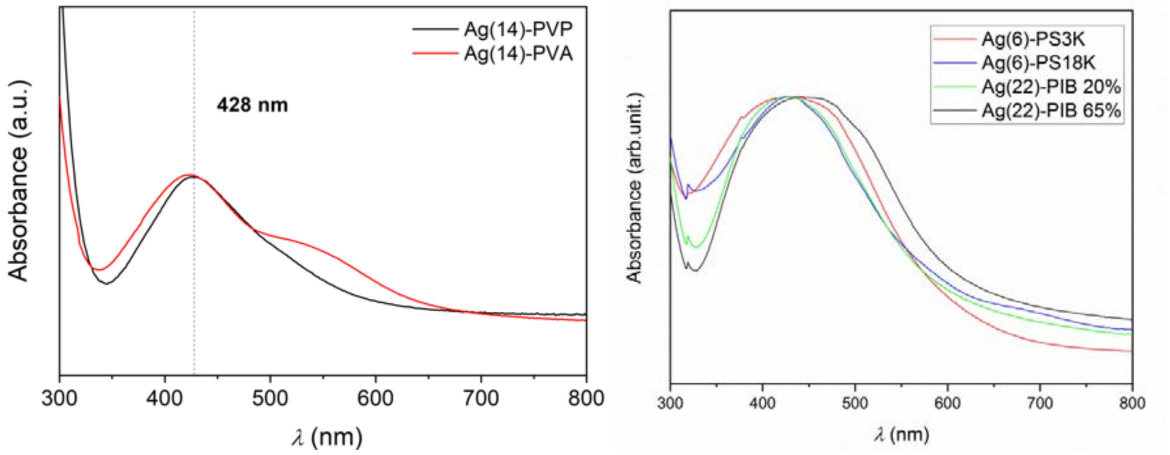


**Figure S2**. UV-vis absorption spectra of dried Ag-PVP and Ag-PVA film (left) and polymer-grafted Ag (right) on glass substrates. The two PS grafts have molecular weight of 3 kDa and 18 kDa, whereas the composition of PIB grafts (with 2.6 kDa) is 20 wt% and 65 wt% metal.

## S3. Composition substrate and matrix effects

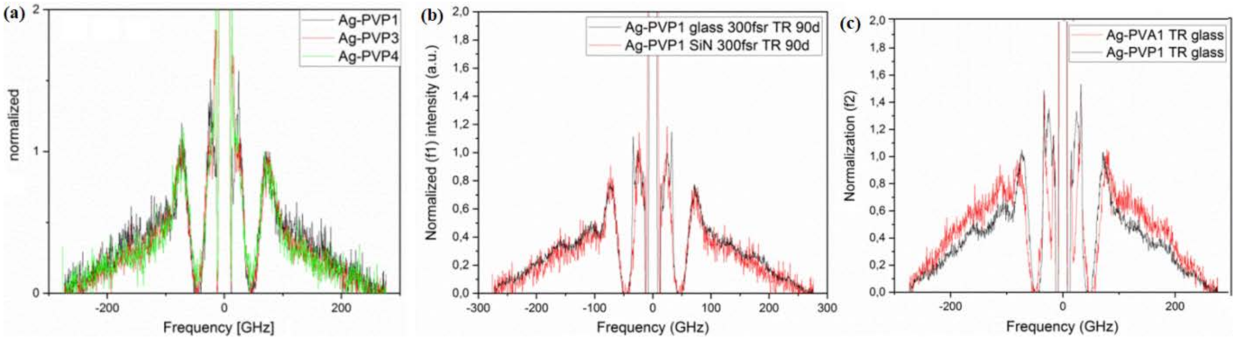


**Figure S3.** (a) Normalized to the peak intensity of the high frequency peak BLS spectra of Ag/PVP nanocomposites. The Ag composition increases from Ag/PVP4 to Ag/PVP1. (b) Supported Ag/PVP1 films on glass and SiN. (c) Glass supported Ag/PVP1 and Ag/PVA films.

## S4. Theoretical modeling of optomechanical coupling

The OM coefficients expressions are obtained based on a first-order perturbation theory [27, 28] and from the knowledge of the acoustic and optical fields in the NP, such as:

$$g_{PE} = -\frac{\omega}{2}\frac{\langle E|\delta\varepsilon|E\rangle}{\iiint_{V_{NP}} \vec{E}.\vec{D}\,dV}\sqrt{\frac{\hbar}{2M_{eff}\Omega}} \quad (1)$$

$$g_{MI} = -\frac{\omega}{2}\frac{\oint_{S_{NP}}(\vec{U}.\vec{n})(\Delta\varepsilon\vec{E}_{\parallel}^{2} - \Delta\varepsilon^{-1}\vec{D}_{\perp}^{2})d\vec{S}}{\iiint_{V_{NP}}\vec{E}.\vec{D}dV}\sqrt{\frac{\hbar}{2M_{eff}\Omega}} \quad (2)$$

Here, $\vec{E}$ is the electric field, $\vec{D}$ is the electric displacement field, $\vec{n}$ is outside normal to the boundary, $\vec{U}$ is the normalized mechanical displacement field associated with the eigenmode of the NP and $\omega$ and $\Omega$ are respectively the optical and acoustic frequencies. The quantity $M_{eff}$ is the effective motional mass which is defined in relation to the (normalized) mechanical displacement field as $M_{eff} = \rho \iiint_{V_{NP}}\|\vec{U}\|^{2}dV$, where $\rho$ is the mass density and $V_{NP}$ is the NP volume.

On the other hand, $\Delta\varepsilon = \varepsilon_1 - \varepsilon_2$, where $\varepsilon_1$ and $\varepsilon_2$ are the dielectric permittivities of the materials on both sides of an interface, and $\delta\varepsilon$ is the amount by which the NP permittivity is deformed due to acoustic strain. In this work, we follow the approach of Ahmed et al consisting in determining the deformed Lorentz-Drude based electric permittivity.[31] The dielectric permittivity of silver can be given by the following Drude-Lorentz expression:

$$\varepsilon_{Ag}(\omega, r) = \varepsilon_r(\infty) + \omega_p^2\sum_{m=1}^{N}\frac{f_m}{\omega_{0m}^2 - \omega^2 - j\omega\Gamma_m} \quad (3)$$

$\omega_p$, is the bulk plasma frequency, $\omega_{0m}$ is the inter-band transition frequency associated with the $m^{th}$ oscillator, $f_m$ is the weight of the latter and $\Gamma_m$ is its relaxation rate. The values of these parameters have been set by fitting equation (3) to experimental data. As the NP goes through the cycle of mechanical compression/expansion, the plasma frequency and inter-band transitions frequencies are deformed. Specifically, electronic density is changed to $N = N_0/(1 + \frac{dV}{V_0})$, where $V_0$ is the initial undeformed NP volume, $dV$ is the change in the latter while the NP deforms and $N_0$ is the initial electronic density; on the other hand we have $\omega_p \propto \sqrt{N}$, hence the change of this parameter. As for the shift in the values of $\omega_{0m}$, they are obtained using deformed potential for silver; the details of such method can be found in the literature.[29-31] Then, the OM coupling rate is defined such as $g_{OM} = g_{MI} + g_{PE}$. The numerical procedure to get the latter consists in first determining the acoustic eigen-modes (and their frequencies) using the same model sketched in Fig. 1(a), then an optical scattering problem is set with the background E-field being defined in PVP medium and linearly polarized. It is worth mentioning that the polarization direction set depends on the investigated mechanical eigen-mode shape. In other words, the polarization is chosen so that a strong overlap between the plasmon and the localized phonon can take place. As a matter of fact, the utilized laser in experiment does not have a specific polarization so that all

directions for the incident E-field have a similar likelihood which means that numerically speaking we can adapt the polarization direction for each mechanical mode under study.

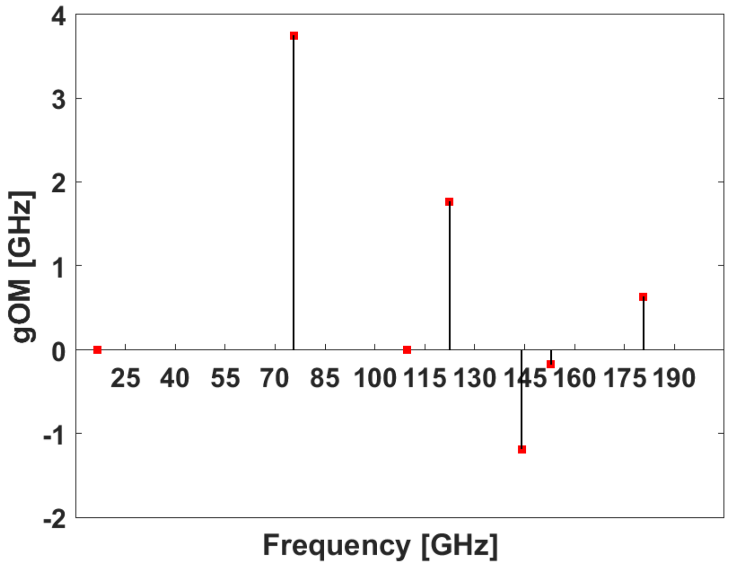


**Figure S4.** Simulated unpolarized OM coefficients versus the frequency for a Ag (14) NP placed in PVP matrix (as shown in Fig. S1a). Note that for the BLS inactive modes $\mathrm{gOM}\ (1) \approx 3.5\mathrm{e}-05\ \mathrm{GHz}$, and $\mathrm{gOM}\ \ (3) \approx 1.8\mathrm{e}-03\ \mathrm{GHz}$ while $\mathrm{gOM}\ (2) \approx 3.7\ \mathrm{GHz}\ (\mathrm{Eg}-\mathrm{mode})$.

**S5: Dimer Ag**

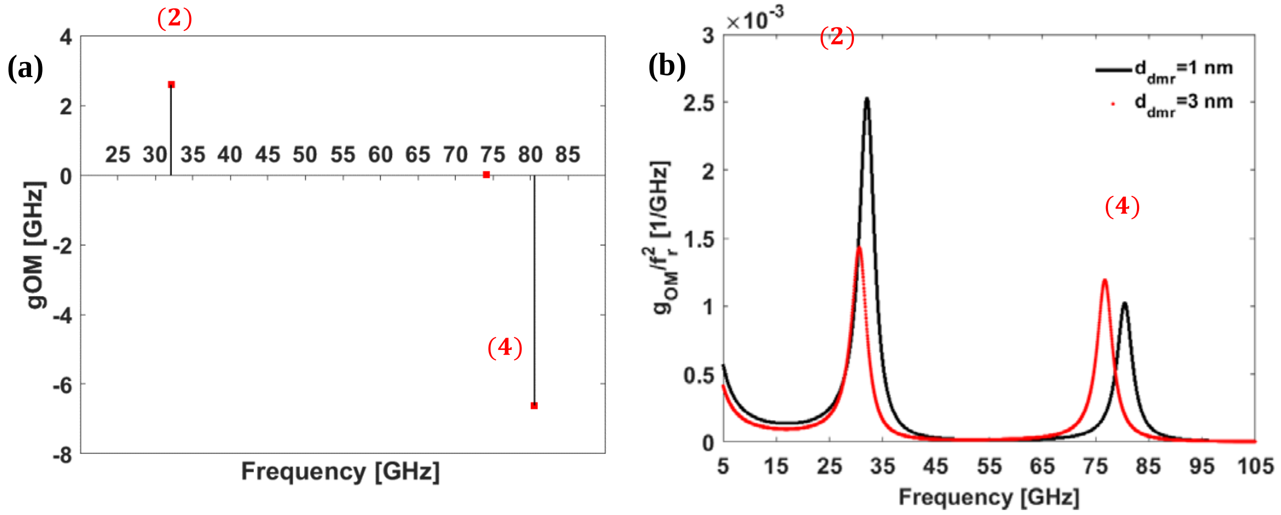


**Figure S5.** (a) Simulated OM coefficients versus frequency for the system depicted in Fig.S1b for $d_{dmr} = 1\ nm$ and such that the incident $\vec{E}$ $-$field oscillates along the dimer axis. (b) Simulated spectrum $g_{\mathrm{OM}}/f^2$ coefficients versus frequency for the Ag dimer/PVP for $d_{dmr} = 1\ nm$ and $d_{dmr} = 3\ nm$ convoluted with a Lorentzian-function having a FWHM equal to $\Delta f$ = 1.5 GHz. In both (a) and (b), rattling and Eg-modes are referred to as (2) and (4), respectively.

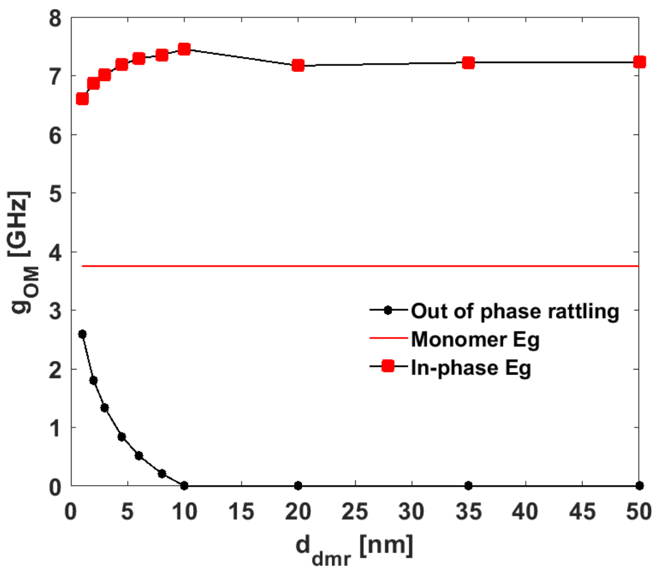


**Figure S6.** Computed OM coefficients associated with the modes referred to as (2) (black line with black doted markers) and (4) (black line with red squared markers) in Figure S5 versus $d_{dmr}$. The red solid line denotes the OM value for the fundamental $Eg$ mode for 14 nm monomer.

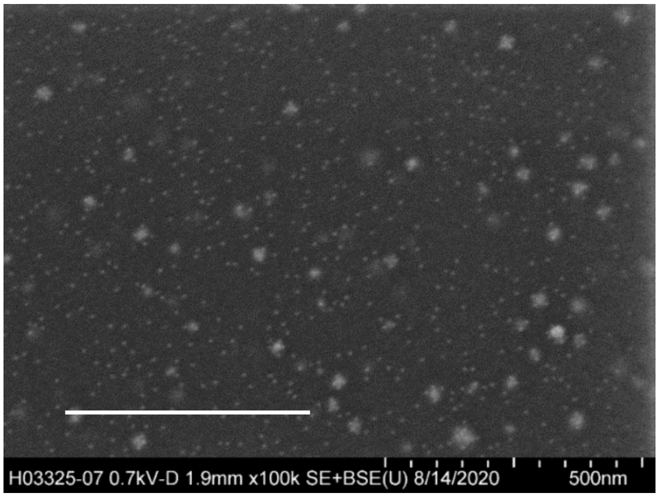


**Figure S7**. SEM image of the Ag (22)/PVP film. The scale bar is 500 nm.

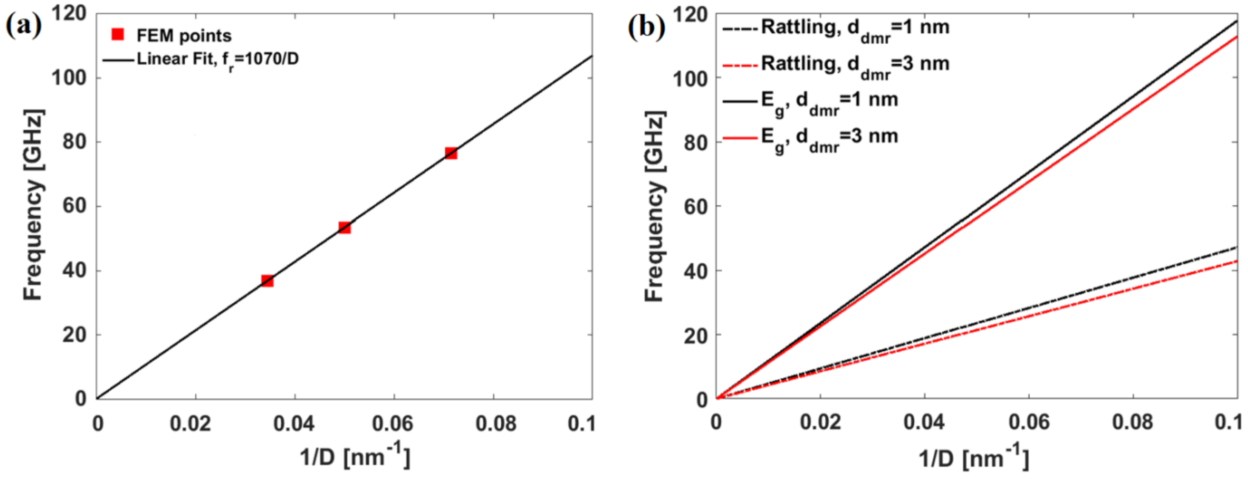


**Figure S8**. Frequency of $E_g$ in the single Ag (a) case and $E_g$ and rattling mode in the case of Ag dimer (b) at two separations in PVA ($C_L$ = 3400 m/s, $C_t$ = 1470 m/s, $\rho$ = 1069 kg/m$^3$) namely, $d_{dmr}$ = 1 and 3 nm. In the case of the dimer at $d_{dmr}$ = 3nm (red lines), the slopes amounts are 1127 m/s and 428 m/s.

**S8: Polymer-grafted Ag NPs (GNP)**

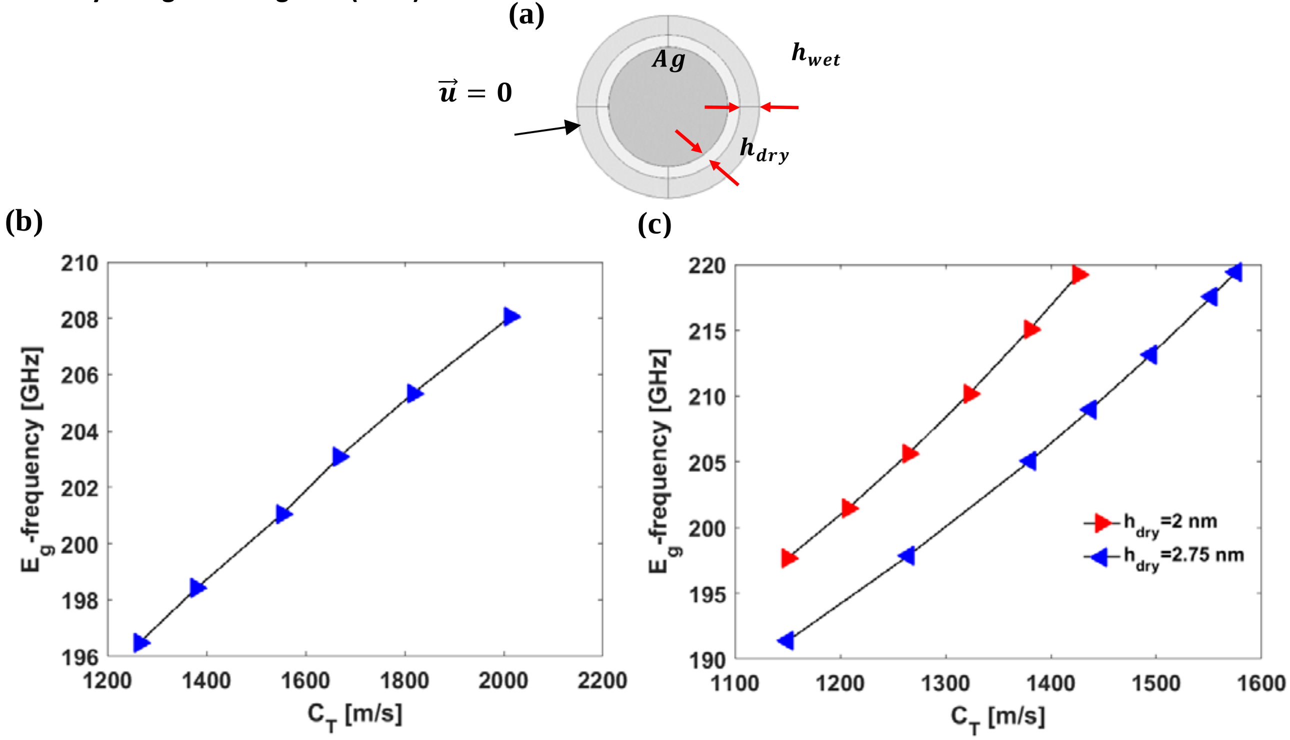


**Figure S9**. (a) Sketch of the employed double layer-core model. (b) Corresponding simulated Eg-frequency versus shear speed (with $C_L = 1.25 \times 2380\ m/s$) of the inner dry region (of 2 nm thickness) while setting the outer interpenetration region (5 nm thickness) to PS elasticity. (c) Same as in (b) but in case where both dry and wet regions shear velocities are simultaneously adjusted. The longitudinal speed of both regions is set as $C_L = 1.25 \times 2380\ m/s$. In both (b) and (c) we constrain the outer interpenetration region layer boundary with the condition, $\vec{u} = \vec{0}$.

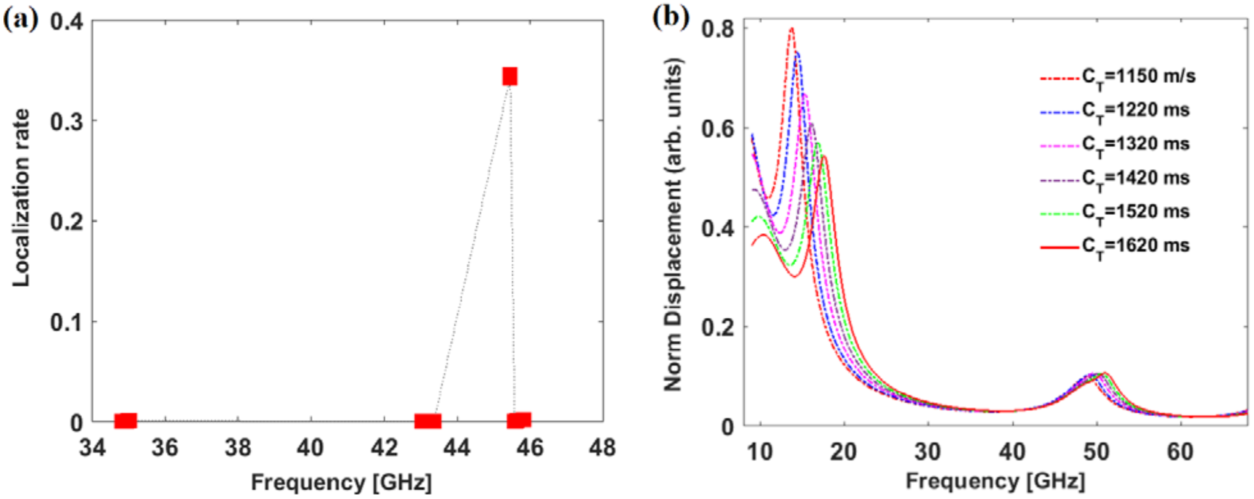


**Figure S10**. (a) Computed eigen-modes localization rate versus frequency in a single Ag (21.6 nm) in liquid PIB ($C_L$ = 2590 m/s; $C_T$ = 0 m/s). This simulation shows the Eg frequency to be situated at $\approx 45.45\ GHz$. (b) The simulated vibration spectrum of a Ag (21.6nm) dimer at distance $d_{dmr} = 5\ nm$ as a function of $C_T$ at constant $C_L$ = 2590 m/s. In addition to Eg mode a rattling mode evolves with increasing PIB rigidity.
